# Supplementary material for: The global speciation continuum of the cyanobacterium Microcoleus
Source: Nat Commun. 2024 Mar 8;15:2122. doi: 10.1038/s41467-024-46459-6 (PMC10923798; doi:10.1038/s41467-024-46459-6)
Supplement: Supplementary file 1 — Supplementary Information [file 41467_2024_46459_MOESM1_ESM.pdf]

Supplementary Information

The global speciation continuum of the cyanobacterium *Microcoleus*

Aleksandar Stanojković[1], Svatopluk Skoupý[1], Hanna Johannesson[2] & Petr Dvořák[1*]

[1] Palacký University Olomouc, Faculty of Sciences, Department of Botany, Šlechtitelů 27, 78371 Olomouc, Czech Republic

[2] The Royal Swedish Academy of Sciences, Department of Ecology, Environment and Plant Sciences, Stockholm University, SE-106 91 Stockholm, Sweden

[*] Corresponding author. E-mail: p.dvorak@upol.cz

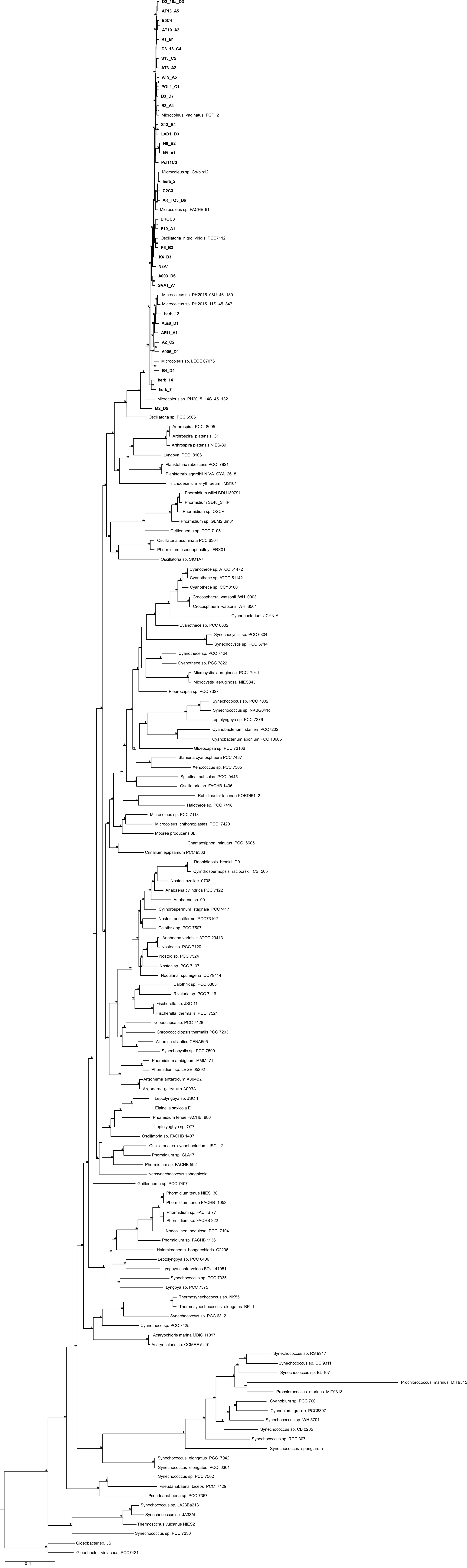

Supplementary Fig. 1. The phylogenetic tree inferred from the maximum likelihood (ML) analysis based on 36 whole-genome sequences of our *Microcoleus* strains (in bold) and 129 other cyanobacteria (dataset 1). *Gloeobacter violaceus* PCC 7421 was u sed as an outgroup. Asterisks at nodes indicate ML bootstrap support of 99 or 100. The scale bar represents substitutions per site.

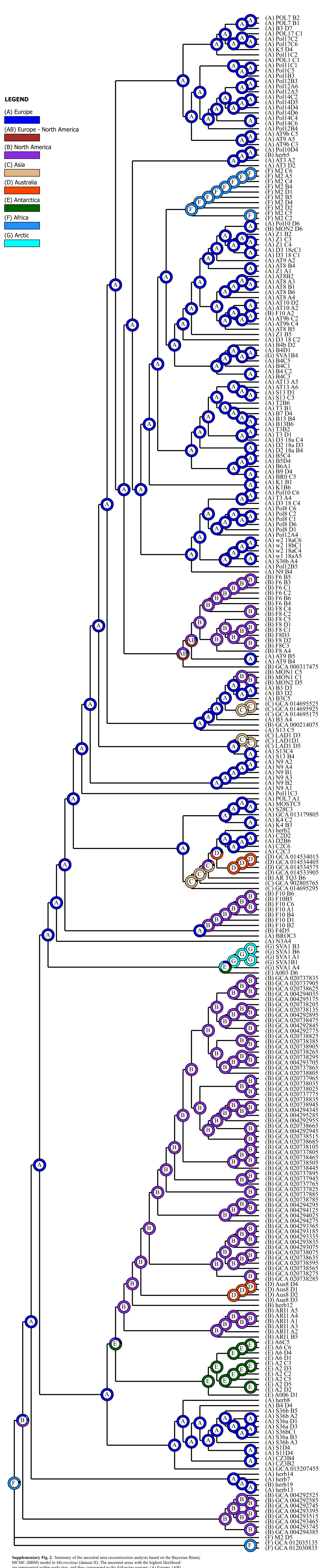

Supplementary Fig. 2. Summary of the ancestral area reconstruction analysis based on the Bayesian Binary MCMC (BBM) model in *Microcoleus* (dataset II). The ancestral areas with the highest likelihood are represented within node pies, and they correspond to the following regions: (A) Europe, (AB) Europe-North America, (B) North America, (C) Asia, (D) Australia, (E) Antarctica, (F) Africa, (G) Arctic.

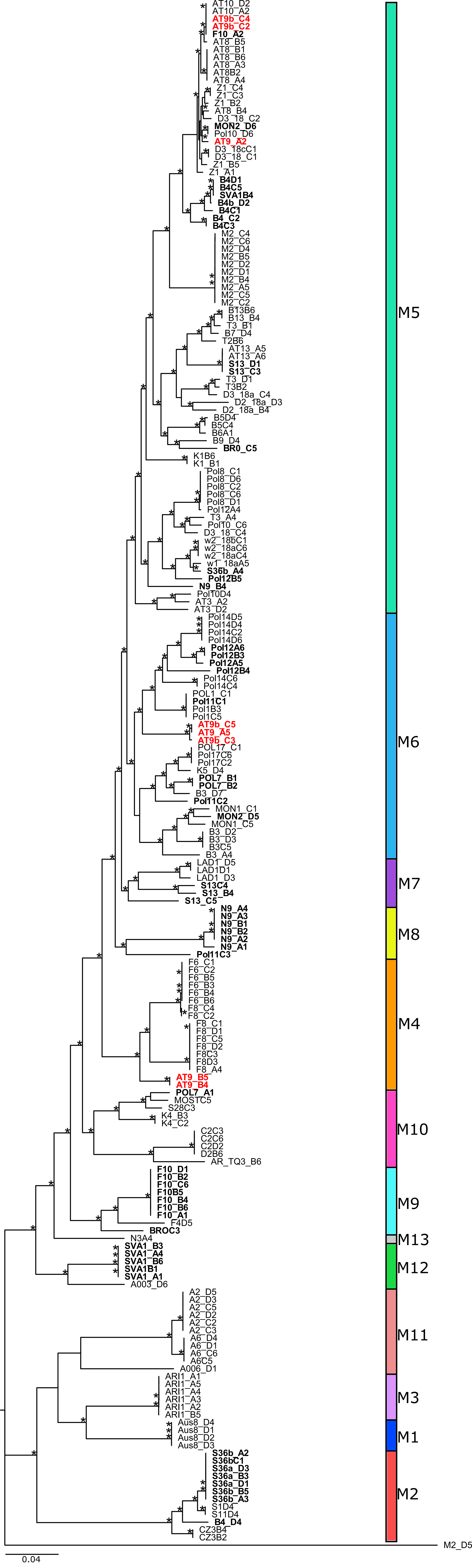

Supplementary Fig. 3. The maximum likelihood (ML) species tree based on 2020 single-copy orthologues of 202 *Microcoleus* strains (dataset III). Strains were clustered into 13 clusters based on the monophyletic criterion and the Bayesian optimized method. Asteri sks at the nodes indicate ML bootstrap support of 99 or 100. Samples containing isolates belonging to two species are indicated in bold, and a sample with isolates belonging to three species is indicated in red. The scale bar represents substitutions per site.

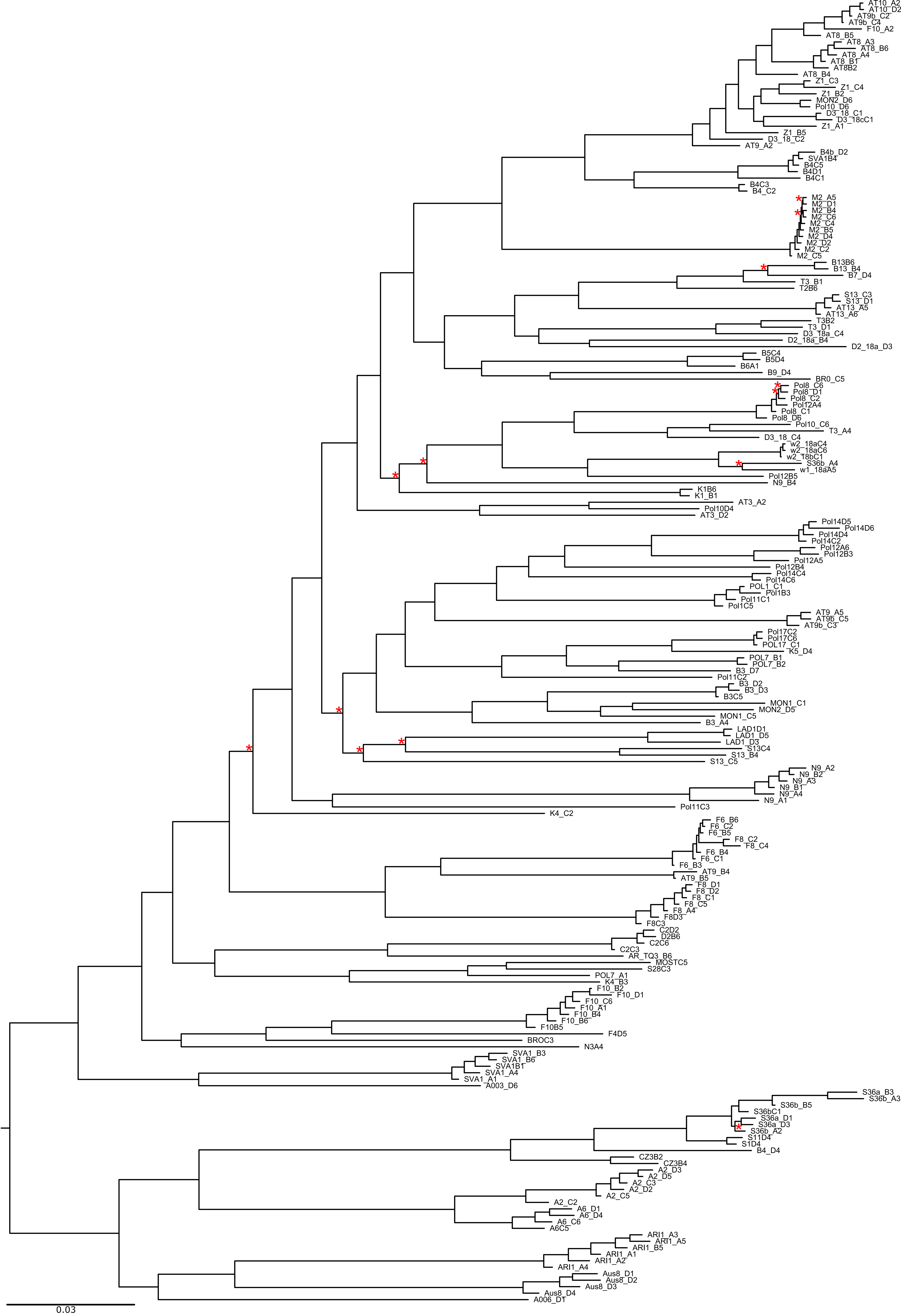

Supplementary Fig. 4. The phylogenetic tree inferred from the maximum likelihood (ML) analysis based on the single nucleotide polymorphisms (SNP) (dataset III, outgroup omitted). Red asterisks at the nodes denote ML bootstrap support less than 99 or 100. The scale bar represents substitutions per site.

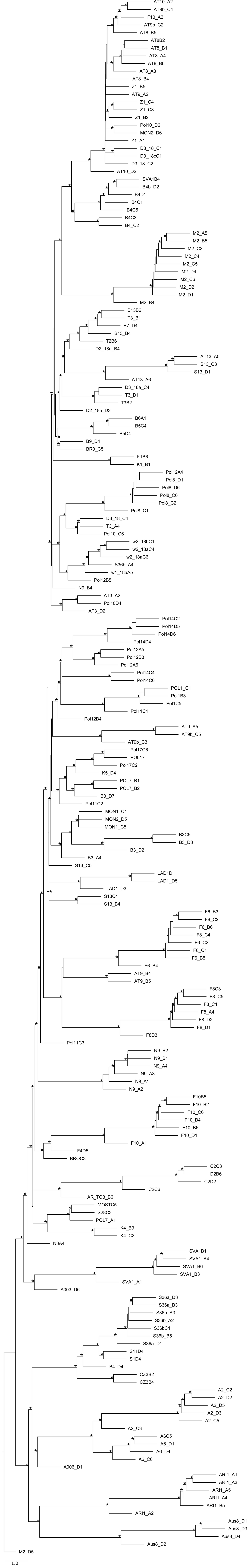

Supplementary Fig. 5. Astral species tree based on individual unrooted gene trees of 2020 single-copy orthologues (dataset III). Asterisks at the nodes represent coalescent units of 1. The scale bar represents substitutions per site.

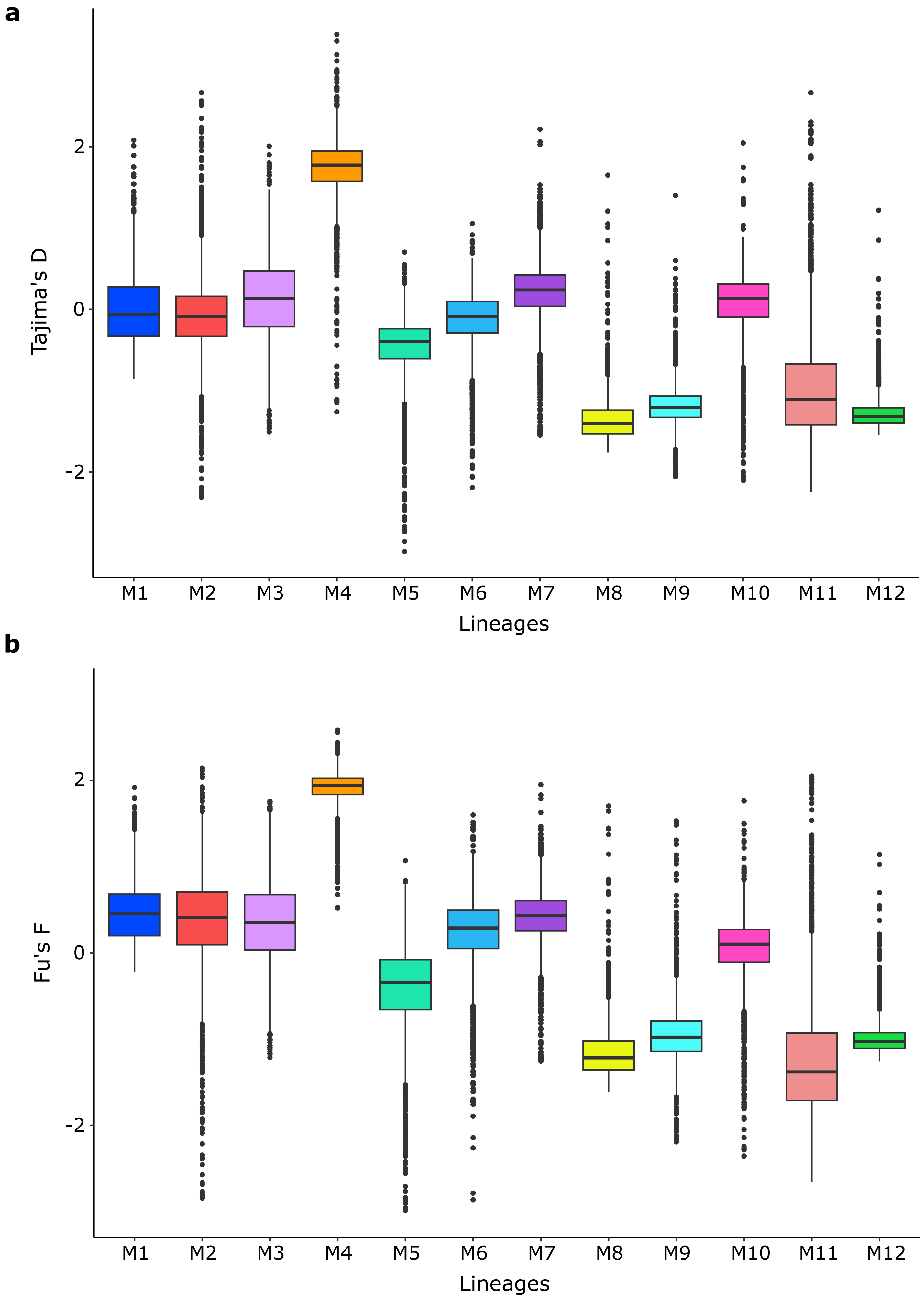

Supplementary Fig. 6. Boxplots representing the distribution of neutrality statistics in 10kb sliding windows with a 2.5k step size for each of the *Microcoleus* lineages (n=200 strains). The colors correspond to the lineages' color codes from Fig. 2. **a** Tajima's D**, b** Fu's F. Boxplots show the median, interquartile range, default whiskers, and outliers as points.

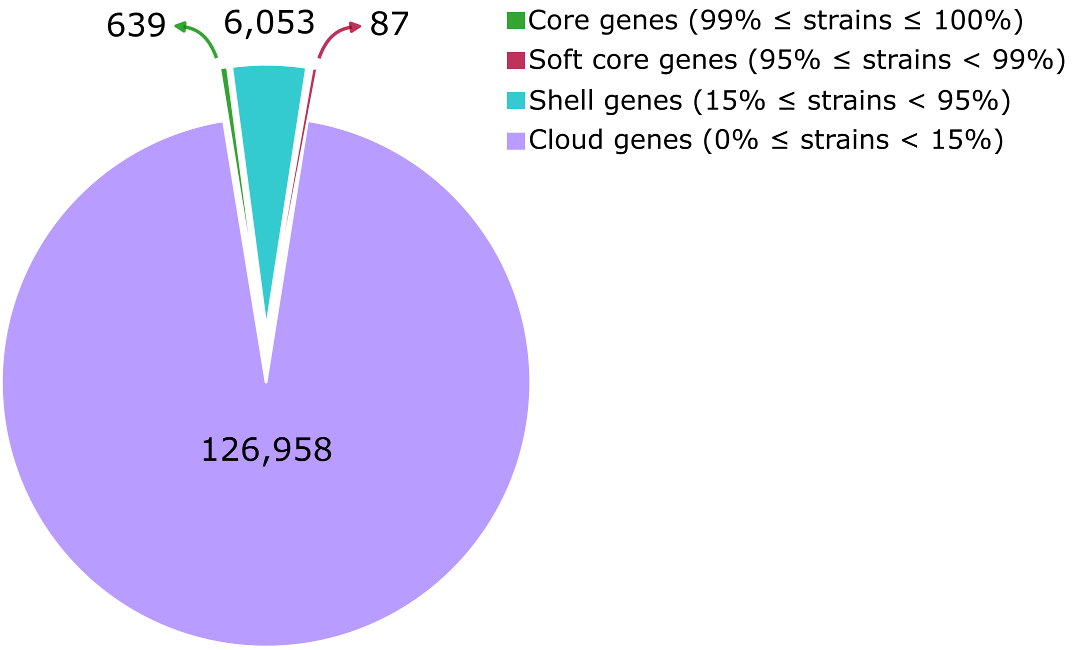

Supplementary Fig. 7. The pangenome of the 201 *Microcoleus* strain is summarized in a pie chart showing the core genes (639), the soft core genes (87), the shell genes (6053), and the cloud genes (126958).

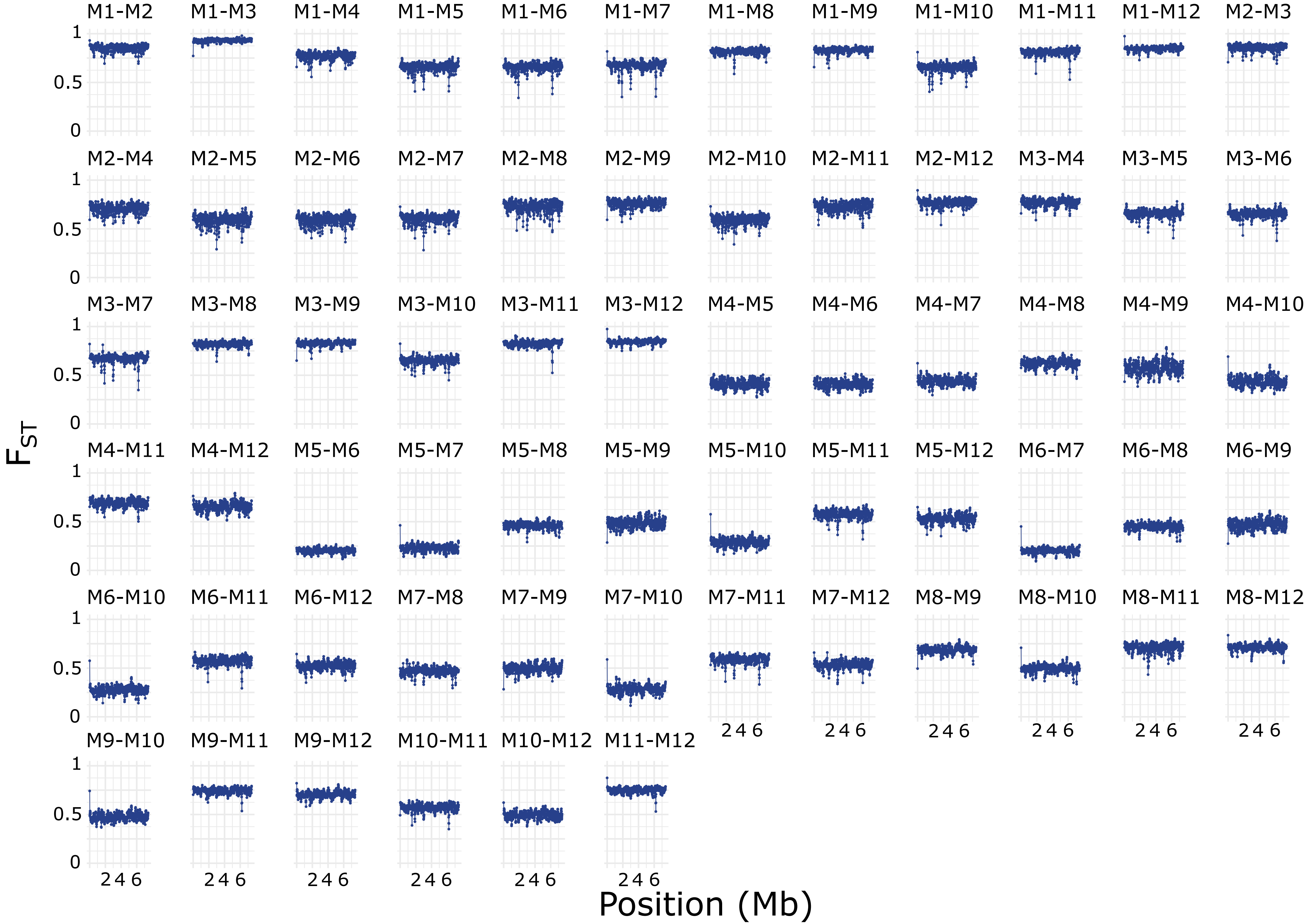

Supplementary Fig. 8. Scatter plots of genomic divergence along the speciation continuum. F_{ST} values were calculated for 50kb windows sliding in increments of 12.5kb. Whole genomes are shown for all pairwise lineage comparisons.

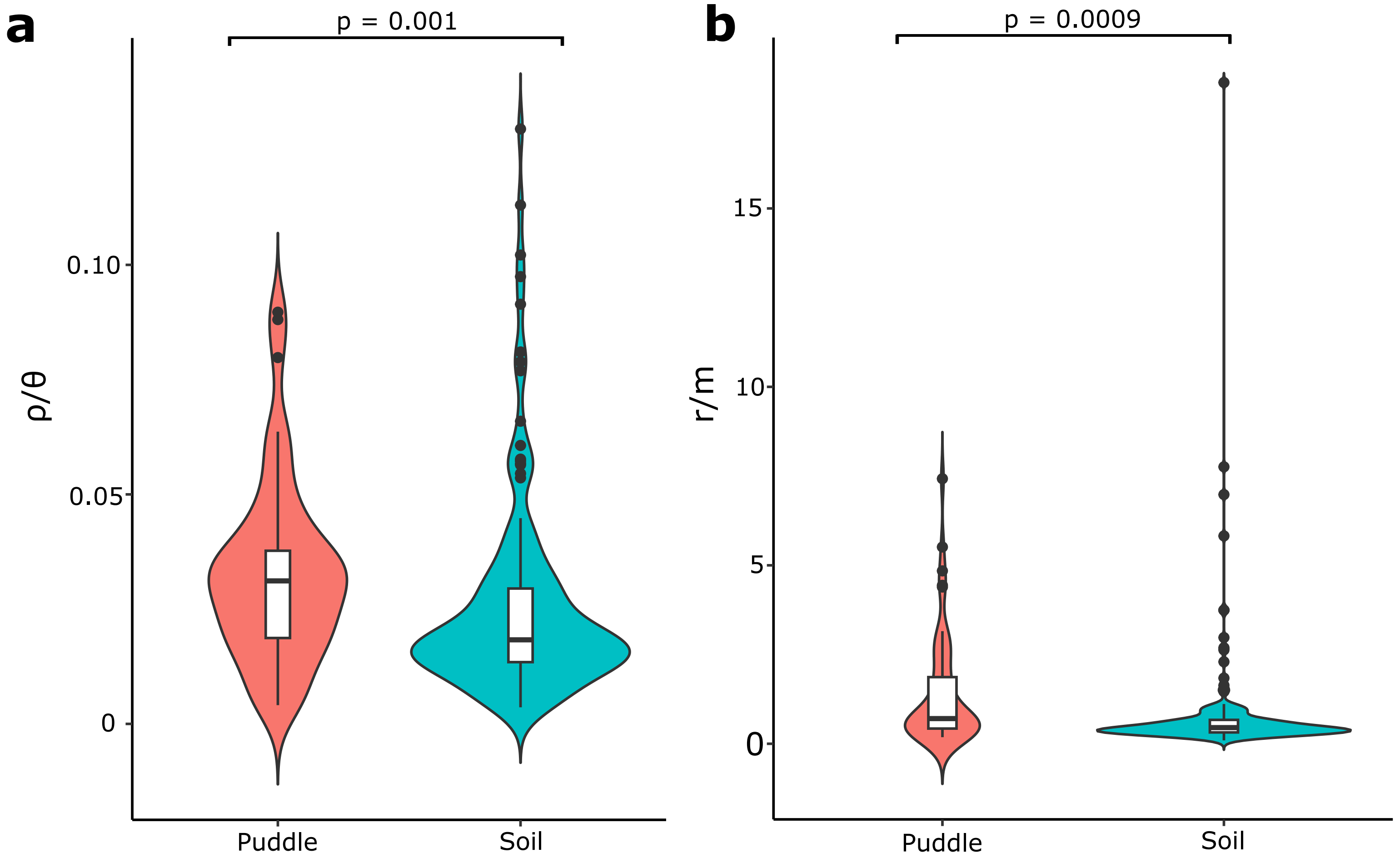

Supplementary Fig. 9. Comparisons of recombination parameters between *Microcoleus* strains occupying puddles and soil (n=201). **a** The violin plot of ρ/θ comparison ($p = 0.001$). **b** The violin plot of r/m comparison ($p = 0.0009$). Statistically significant Kruskal-Wallis test is indicated on the top bar. Boxplots show the median, interquartile range, default whiskers, and outliers as points.

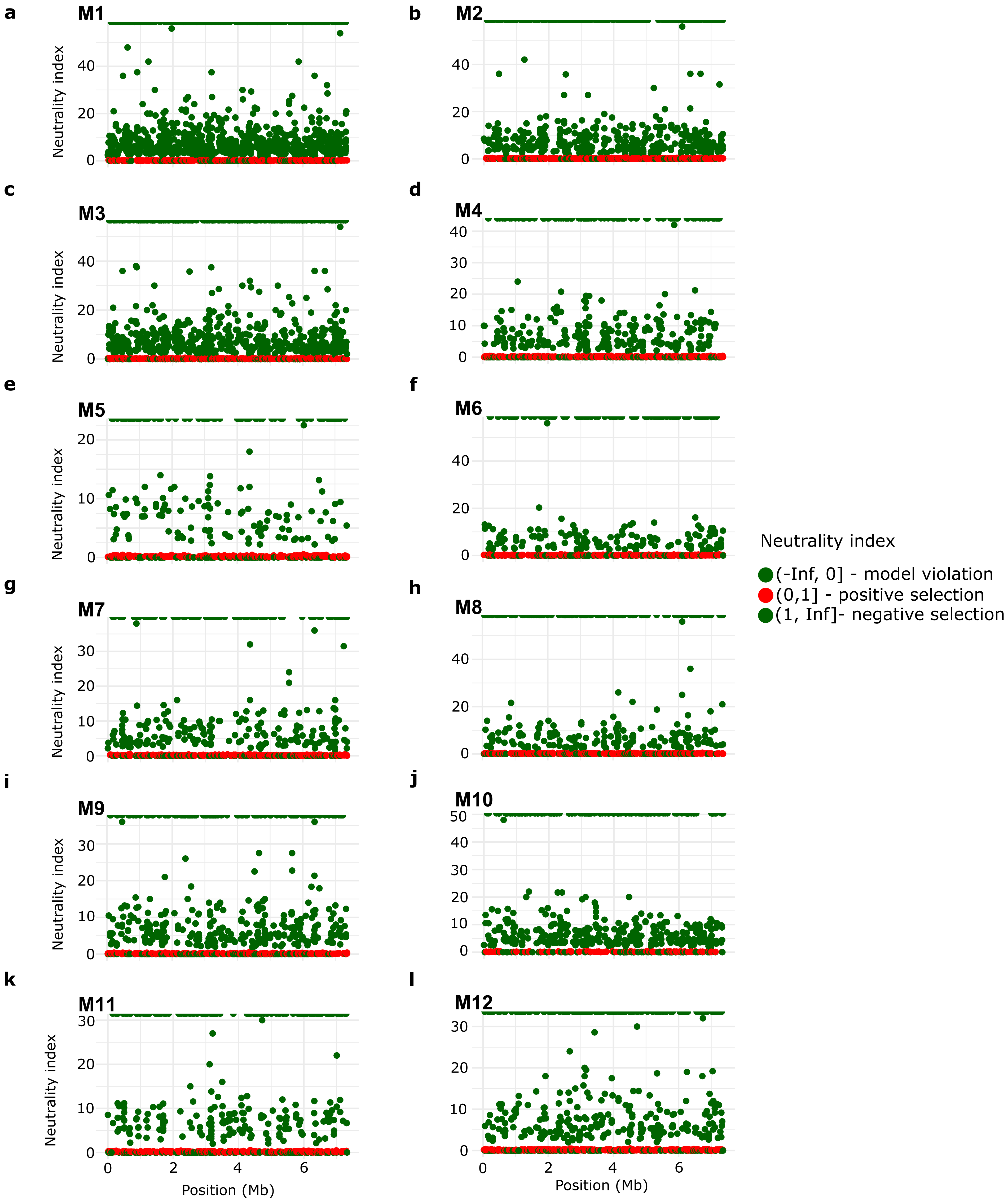

Supplementary Fig. 10. The distribution of the neutrality index (NI) across the genome per *Microcoleus* lineage. All the NI values per lineage were mapped over the reference genome *Oscillatoria nigro-viridis* PCC 7112. NI values lower than 1 indicate positive selection (red), and those higher than 1 indicate negative selection. Figures from a-l correspond to *Microcoleus* lineages from M1-M12.
