# Supplementary material for: The global speciation continuum of the cyanobacterium Microcoleus
Source: Nat Commun. 2024 Mar 8;15:2122. doi: 10.1038/s41467-024-46459-6 (PMC10923798; doi:10.1038/s41467-024-46459-6)
Supplement: Supplementary file 3 — Description of Additional Supplementary Files [file 41467_2024_46459_MOESM3_ESM.pdf]

Description of Additional Supplementary Files:

Supplementary Data 1: Genomic and geographic features of *Microcoleus* strains included in this study (asterisk marks genomes used in dataset I).

Supplementary Data 2: Taxa list with GenBank accession numbers used in datasets I and II.

Supplementary Data 3: A number of lineages found in environmental samples of *Microcoleus*.

Supplementary Data 4: The resulting clustering of *Microcoleus* strains inferred by the ANI, fastBAPS, snapclust, and the GTDB-Tk.

Supplementary Data 5: The pairwise comparison of average nucleotide identity (ANI) between *Microcoleus* strains (dataset III).

Supplementary Data 6: Tajima's D and Fu's F neutrality statistics per window over the wholegenome in a 10kb window and 5kb step size.

Supplementary Data 7: The list of genes found to be significantly associating and disassociating in the *Microcoleus* pangenome predicted by Coinfinder.

Supplementary Data 8: A number of HGT events per taxon donor as predicted by HGTector2. Only the top 15 taxons are represented in Fig. 3a.

Supplementary Data 9: A number of HGT events per strain predicted by HGTector2. These values are shown in Fig. 3c and 3d.

Supplementary Data 10: Dunn's test of significant differences between pairs of lineages in the HGT rates per Mb shown in Fig. 3c. The asterisk indicates statistically significant pairwise lineage comparisons.

Supplementary Data 11: Values of nucleotide diversity (nd), the fixation index (F_{ST}), and the absolute divergence (D_{XY}) over the whole genome in 50kb window size and 12.5kb step size for 12

Microcoleus lineages (regions of exceptional divergence - 99th percentile of both F_{ST} and D_{XY} are colored orange).

Supplementary Data 12: Dunn's test of significant differences between pairs of lineages in the environmental variables present in their habitats shown in Fig. 2c-2f. The asterisk indicates statistically significant pairwise lineage comparisons.

Supplementary Data 13: Phylogenetic signal and Mantel test results for environmental variables. Highlighted in red are statistically significant Mantel tests and highlighted in yellow are variables represented in Fig. 2.

Supplementary Data 14: Genome fractions subjected to recombination shared between strains of the same lineage (within) and between strains of different lineages (outside) calculated from the Gubbins output (recombination_predictions.gff file). These values are represented in Figs. 4a and 4b.

Supplementary Data 15: The extent of gene flow between and within lineage pairs (in %) and the values represented in Figs. 4a and 4b calculated from the Gubbins output.

Supplementary Data 16: Genome fractions resistant to gene flow between *Microcoleus* lineages derived from Supplementary Data 15 for defining species according to the UPCEL.

Supplementary Data 17: Recombination parameters per strain calculated by Gubbins.
