# Supplementary material for: The global speciation continuum of the cyanobacterium Microcoleus
Source: Nat Commun. 2024 Mar 8;15:2122. doi: 10.1038/s41467-024-46459-6 (PMC10923798; doi:10.1038/s41467-024-46459-6)
Supplement: Supplementary file 5 — Reporting Summary [file 41467_2024_46459_MOESM5_ESM.pdf]

nature portfolio

Corresponding author(s): Petr Dvorak
Last updated by author(s): Nov 24, 2023

Reporting Summary

Nature Portfolio wishes to improve the reproducibility of the work that we publish. This form provides structure for consistency and transparency in reporting. For further information on Nature Portfolio policies, see our Editorial Policies and the Editorial Policy Checklist.

Statistics

For all statistical analyses, confirm that the following items are present in the figure legend, table legend, main text, or Methods section.

n/a	Confirmed	
☐	☒	The exact sample size (*n*) for each experimental group/condition, given as a discrete number and unit of measurement
☐	☒	A statement on whether measurements were taken from distinct samples or whether the same sample was measured repeatedly
☐	☒	The statistical test(s) used AND whether they are one- or two-sided<br>*Only common tests should be described solely by name; describe more complex techniques in the Methods section.*
☒	☐	A description of all covariates tested
☒	☐	A description of any assumptions or corrections, such as tests of normality and adjustment for multiple comparisons
☐	☒	A full description of the statistical parameters including central tendency (e.g. means) or other basic estimates (e.g. regression coefficient) AND variation (e.g. standard deviation) or associated estimates of uncertainty (e.g. confidence intervals)
☐	☒	For null hypothesis testing, the test statistic (e.g. *F*, *t*, *r*) with confidence intervals, effect sizes, degrees of freedom and *P* value noted<br>*Give P values as exact values whenever suitable.*
☐	☒	For Bayesian analysis, information on the choice of priors and Markov chain Monte Carlo settings
☒	☐	For hierarchical and complex designs, identification of the appropriate level for tests and full reporting of outcomes
☐	☒	Estimates of effect sizes (e.g. Cohen's *d*, Pearson's *r*), indicating how they were calculated

Our web collection on statistics for biologists contains articles on many of the points above.

Software and code

Policy information about availability of computer code

Data collection	No software was used for data collection.
Data analysis	Trimmomatic v0.39, SPAdes v3.13.1, MaxBin v2.2.4, CheckM v1.1.9, prokka v1.14.5, freebayes v1.3.2, snippy v4.6, Roary v3.13.0, Coinfinder v1.0.7, HGTector v2.0b3 , R 4.1.3, Orthofinder v2.3.1, IQ-TREE v1.6.1, ASTRAL-III, R package fastBAPS, R package adegenet v2.1.5, fastANI v1.33, GTDB-Tk v2.3.2 , BEAST v1.10.4, RASP v4.3, R package geosphere v1.5-18, R package vegan v2.5.6, R package dunn.test v1.3.5, R package picante v1.8.2, R package Geiger v2.0.1, , Gubbins v3.1.3, RAxML-NG, Phandango v1.3.0, R packages reshape2 v1.4.4, PopGenome v2.7.5, R package ggplot2 v3.3.5, BEAST2 v2.7.4, eggNOG-mapper v2.1.12, scripts: https://github.com/dvorikus/Microcoleus-population-genomics

For manuscripts utilizing custom algorithms or software that are central to the research but not yet described in published literature, software must be made available to editors and reviewers. We strongly encourage code deposition in a community repository (e.g. GitHub). See the Nature Portfolio guidelines for submitting code & software for further information.

Data

Policy information about availability of data

All manuscripts must include a data availability statement. This statement should provide the following information, where applicable:

- Accession codes, unique identifiers, or web links for publicly available datasets
- A description of any restrictions on data availability
- For clinical datasets or third party data, please ensure that the statement adheres to our policy

Biosample identification and accession numbers are available in Supplementary Data 1. Previously published whole genome sequences are available under the GenBank accession numbers listed in Supplementary Data 1 and 2. Multiple sequence alignments and datasets used and/or analyzed during the study can be found in Supplementary Data 1-17 as well as at https://doi.org/10.6084/m9.figshare.24710961.v1 and https://github.com/dvorikus/Microcoleus-population-genomics. The environmental variables were extracted from the following databases: WorldClim (https://www.worldclim.org/), ISRIC SoilGrids (www.isric.org), glUV (https://www.ufz.de/gluv/), and HANPP database (https://sedac.ciesin.columbia.edu/data/collection/hanpp).

Research involving human participants, their data, or biological material

Policy information about studies with human participants or human data. See also policy information about sex, gender (identity/presentation), and sexual orientation and race, ethnicity and racism.

Reporting on sex and gender	NA
Reporting on race, ethnicity, or other socially relevant groupings	NA
Population characteristics	NA
Recruitment	NA
Ethics oversight	NA

Note that full information on the approval of the study protocol must also be provided in the manuscript.

Field-specific reporting

Please select the one below that is the best fit for your research. If you are not sure, read the appropriate sections before making your selection.

☐ Life sciences ☐ Behavioural & social sciences ☒ Ecological, evolutionary & environmental sciences

For a reference copy of the document with all sections, see nature.com/documents/nr-reporting-summary-flat.pdf

Ecological, evolutionary & environmental sciences study design

All studies must disclose on these points even when the disclosure is negative.

Study description	This study includes a global collection of environmental samples (soils and puddles) containing the cyanobacterium Microcoleus. Unialgal cultures were established from these samples and subsequently underwent Illumina sequencing to acquire whole-genome sequences. A total of 210 genomes was acquired and then used for population genomic analyses to investigate the continuum of Microcoleus populations and possible genetic factors associated with their dominance in terrestrial ecosystems. The study further explored the impact of various evolutionary factors on the divergence of Microcoleus lineages as well as signatures of local selection and gene flow over the whole genome during adaptive divergence.
Research sample	Our study encompasses newly developed datasets based on genomes sequenced here as well as genome sequences obtained from the GenBank database (accession numbers available in Supplementary Data 1 and 2). Altogether, 76 samples of soil and puddles from all continents except South America were gathered. Then, from each sample, we isolated 1-11 strains of the cyanobacterium Microcoleus spp., reaching 495 strains altogether. To confirm that strains belong to Microcoleus, we sequenced 16S rRNA and 16S-23S ITS and found 13 monophyletic clades/lineages that might represent novel species. Thus, our previous study served as a priori information on the population genetic structure of Microcoleus lineages (10.1080/09670262.2021.2007420). The selection of 202 strains for genome sequencing in this study followed guidelines for population genomic and general reverse ecology approaches (e.g., 10.1016/j.cell.2019.06.033; 10.1101/cshperspect.a018143; 10.1016/j.algal.2023.103128).<br><br>Our sampling design aimed to capture a diverse array of genotypes belonging to different Microcoleus spp. lineages. Hence, our samples constitute a representative subset of the Microcoleus lineages on a global scale.
Sampling strategy	Collections of Microcoleus spp. were made during 2019. Sampling locations had diverse climates and habitats: soil (top layer, ~2cm upper), puddles (ephemeral concave water bodies, 5-10cm depth), and moss vegetation (belowground parts). The samples were collected with a sterile spatula and placed directly in a sterile plastic bag. They were stored in a dark and cool place during transport. Once in the lab, small quantities of each sample were placed directly in 10 ml capped tubes with Zehnder medium. Part of the grown

	biomass was then transferred to Petri dishes on solidified agar Zehnder medium. From each environmental sample, one to 11 unialgal cultures were obtained. All environmental samples are stored at 4 °C and the cultures are maintained at the Department of Botany, Palacký University in Olomouc at 22 ± 1 °C, illuminated with an average photon flux density of 20 µmol photons m-2 s−1, and under regime 12 h light/12 h dark.<br><br>Given the lack of explicit evaluations regarding minimal sample size for bacterial populations, our approach involved sequencing genomes from as many Microcoleus isolates as possible within each of the 13 monophyletic lineages. The selection of strains was such to reconstruct the population genetic structure based on a previous study (10.1080/09670262.2021.2007420). The lineages varied in size, ranging from 4 to 149 strains. Consequently, we sequenced whole genomes to ensure a sufficient representation of each lineage for population genomic analyses.
Data collection	Approximately 100mg of fresh biomass was used for extracting the genomic DNA of Microcoleus strains. Genomes were sequenced on the Illumina NovaSeq 6000 platform at the 150 bp x 2 paired-end mode. All the reads were trimmed, filtered, and then assembled into whole genomes. In total, there were 202 whole genomes.<br><br>Eight Microcoleus vaginatus samples were obtained from the herbarium of the Natural History Museum in London, United Kingdom. The samples were extracted following Kistler's protocol (see main text) and then sequenced on the Illumina platform.<br><br>In this study, we sequenced 210 Microcoleus genomes altogether.<br><br>Additionally, in order to fully encompass the wide range of ecological factors and geographic locations where this cyanobacterium thrives, improving phylogenomic analyses as well as statistics, we obtained 81 Microcoleus genomes from the GenBank database (see Supplementary Data 2 for accession numbers). In that way, we constructed three datasets: (1) Dataset I with 165 genomes for phylogenomic analyses; (2) Dataset II with 291 genomes for phylogenomic analyses, ancestral area reconstruction, Mantel and phylogenetic signal tests; (3) Dataset III with 202 genomes for phylogenomic, dating, delimitation, pangenome, variant calling, HGT, and population genetic analyses.
Timing and spatial scale	The spatial scale of this study is global, including samples from all continents apart from South America.<br><br>Field sampling of Microcoleus was performed between 2015 and 2019 (detailed information on the sampling is in Stanojkovic´et al. 2022, Eur. J. Phycol. https://doi.org/10.1080/09670262.2021.2007420). Herbarium specimens were deposited between 1825 and 1938. The samples were collected once from each of the localities.
Data exclusions	No data were excluded from the analyses.
Reproducibility	The list of accession numbers for all genomes used in this study are available in Supplementary Data 1 and 2. Details about the datasets and codes used can be found at https://doi.org/10.6084/m9.figshare.24710961.v1 and https://github.com/dvorikus/Microcoleus-population-genomics.
Randomization	No experiment required group assignment of individuals.
Blinding	Blinding strategy is not relevant for this study.

Did the study involve field work? ☐ Yes ☒ No

Reporting for specific materials, systems and methods

We require information from authors about some types of materials, experimental systems and methods used in many studies. Here, indicate whether each material, system or method listed is relevant to your study. If you are not sure if a list item applies to your research, read the appropriate section before selecting a response.

Materials & experimental systems

n/a	Involved in the study	
☒	☐	Antibodies
☒	☐	Eukaryotic cell lines
☒	☐	Palaeontology and archaeology
☒	☐	Animals and other organisms
☒	☐	Clinical data
☒	☐	Dual use research of concern
☒	☐	Plants

Methods

n/a	Involved in the study	
☒	☐	ChIP-seq
☒	☐	Flow cytometry
☒	☐	MRI-based neuroimaging

Plants

Seed stocks	Not applicable
Novel plant genotypes	Not applicable
Authentication	Not applicable
